# Kinetics of elastic recovery in roll compaction

**DOI:** 10.1016/j.ijpx.2024.100303

**Published:** 2024-11-14

**Authors:** Martin Lück, Stefan Klinken-Uth, Peter Kleinebudde

**Affiliations:** Heinrich Heine University Düsseldorf, Faculty of Mathematics and Natural Sciences, Institute of Pharmaceutics and Biopharmaceutics, Universitaetsstrasse 1, 40225 Düsseldorf, Germany

**Keywords:** Roll compaction, Elastic recovery, Kinetics, Viscoelasticity

## Abstract

Elastic recovery (ER) has been investigated and discussed extensively in the field of tableting. However, until now only limited data is available regarding ER in roll compaction. Therefore, a previously established in-line measurement technique was rolled out to further investigate the kinetics of ER in roll compaction and the effects of specific compaction force (SCF) and roll speed (RS). In-line laser triangulation measurements at different positions within a roll rotation as well as measurement over time after the process has been stopped were utilized. Pure microcrystalline cellulose (MCC) and two placebo powder blend formulations were analysed. Successful fit of the contained ER profiles emphasized that the ER on the roll surface is build out of two exponential kinetics. Starting with a dominating fast ER (${\mathrm{ER}}_{A}$), characterized by a high increase of the ribbon thickness after passing the gap width, followed by a slower ER (${\mathrm{ER}}_{B}$). Sigma minus plot analysis showed that increasing RS led to an accelerated ${\mathrm{ER}}_{A}$ and ${\mathrm{ER}}_{B}$ which was related to the viscoelastic behaviour of MCC. The SCF only had an effect on the kinetics of ER if a brittle filler was added to the mixture. The conducted study established the first approach in literature to characterize the kinetics of ER in roll compaction. It supports the understanding and characterization of relaxation times and the effect of the RS and SCF in roll compaction.

## Highlights

• First in-line approach to investigate elastic recovery kinetics in roll compaction.
• Elastic recovery on the roll surface follows two different exponential kinetics.
• Sigma minus plot showed increased roll speed accelerated elastic recovery for MCC.
• Specific compaction force affected recovery only with brittle filler added.

## Graphical abstract

**Figure:**
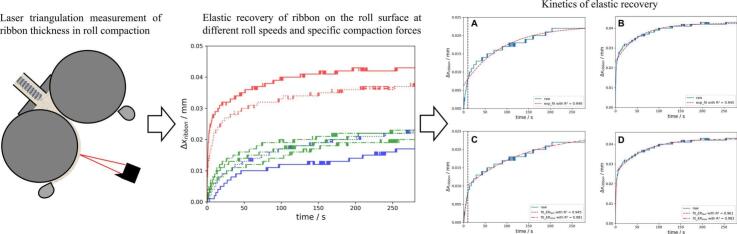

## 1 Introduction

### 1.1 Roll compaction and introduction of elastic recovery

Roll compaction is an established technique in the production of oral solid dosage forms and described detailed in the literature over many years (Jaminet and Hess, 1966; Kleinebudde, 2004, Kleinebudde, 2022; Miller, 2005; Reynolds et al., 2010; Souihi et al., 2015; Sun and Kleinebudde, 2016). After the powder is densified, the highest ribbon solid fraction (${\mathrm{SF}}_{\mathrm{gap}}$) is reached at the minimum distance between both counter rotating rolls, described as gap width (S). The stages of compaction were in generally postulated by Train (Train, 1956). After the powder volume is reduced to the minimum volume, which happens at S, the pressure is released and the elasticity of the material predominates. This leads to elastic recovery (ER) as the thickness of the compact increases (Keizer and Kleinebudde, 2020; Train, 1956) and therefore the solid fraction decreases until ${\mathrm{SF}}_{\mathrm{ribbon}}$ is reached. Due to pressure distribution over the roll width (W) in roll compaction the ribbon thickness and ${\mathrm{SF}}_{\mathrm{ribbon}}$ is not uniform (Krok and Wu, 2019; Michrafy et al., 2017; Souihi et al., 2015). This is highly influenced by the sealing system used in roll compaction. While rim rolls cause a more uniform pressure distribution and ribbon density ($\rho_{\mathrm{ribbon}}$), cheek plates result in a heterogeneous pressure distribution (Mazor et al., 2016) which may effect ER.

The extent of ER is influenced by the material properties. Therefore, deformation behaviour under compaction can be mainly differentiated into three categories: elastic, plastic and fragmentation. However, pharmaceutical materials cannot be assigned exclusively to one of these deformation behaviours. Therefore the specific properties of a material vary to a certain extent between elastic, plastic and brittle (Antikainen and Yliruusi, 2003; Roberts and Rowe, 1987). Plastic flow and brittle fragmentation are important to build bonds between particles and lead to ribbons of a certain strength and density which influences the particle size distribution after milling (Souihi et al., 2013). On the other hand, elasticity can cause issues like splitting of the ribbons (Mahmah et al., 2019) or capping and lamination of tablets (Paul and Sun, 2017). However, knowledge of the elastic deformation after compaction is crucial to successfully predict ${\mathrm{SF}}_{\mathrm{ribbon}}$. This was shown in a previous study using in-line laser triangulation measurement in roll compaction (Luck et al., 2024) and in the hybrid model approach using uniaxial compaction simulation by Keizer and Kleinebudde (Keizer and Kleinebudde, 2020).

### 1.2 Investigation of elastic recovery in tableting and roll compaction

Overall, ER can be divided into the spontaneous, fast in-die ER and the time-dependent, slow ER after the ejection of the tablet (Haware et al., 2010). As all modern compaction simulators are instrumented with force and displacement sensors, which enables time saving in-die analysis, the majority of knowledge about ER was gained in tableting or uniaxial compaction simulation experiments and correlates quite well with out-of-die data (Katz et al., 2013). It was postulated that ER can weaken the strength of compacts (Armstrong and Haines-Nutt, 1972; Esezobo and Pilpel, 1986), which might be overcome by granulation of the powder blend to ensure sufficient tensile strength of the tablets (Armstrong and Haines-Nutt, 1972). However, ER can also be connected to lamination of tablets. For this reason Dwivedi et al. calculated the in-die ER with a direct relationship between the compression force and machine deformation to successfully estimate the Young's modulus of the used materials (Dwivedi et al., 1992). Moreover in-die ER was found to be an effective parameter to detect air entrapment in tablets (Vreeman and Sun, 2022). Following this strategy the pre-compression pressure can be optimized by selecting it to the minimum in-die ER, which indicated the lowest amount of entrapped air and therefore reduced the risk of lamination (Vreeman and Sun, 2024). In contrary to the field of tableting only few publications about the ER in roll compaction itself are available because no industrial available roll compactor is instrumented with pressure sensors which makes the analysis of the pressure curve impossible. In addition, the measurement of the roll displacement is not useful as rolls do not move in repeating cycles like punches in tableting. Moreover the thickness measurement of the ribbons out-of-die might be challenging due to the weakness or lamination of ribbons, which can result in misleading findings. This leads to a lack of understanding ER in roll compaction process.

### 1.3 Influence of the pressure on elastic recovery

The influence of compression pressure is widely discussed in literature. Train postulated that the ER is independent of any compression pressure. In addition he emphasized that full ER can only take place after ejection of the compacts (Train, 1956). In contrary to Train, Mahmah et al. stated that higher hydraulic pressure led to an increase in ER for different kinds of pharmaceutical powders like microcrystalline cellulose (Picker, 2001), mannitol or lactose (Mahmah et al., 2019). Other authors showed that for plastic materials like microcrystalline cellulose and maize starch a minimum in ER could be observed between 90 and 150 MPa. Afterwards the ER increases again (Antikainen and Yliruusi, 2003; Arndt and Kleinebudde, 2018; Keizer and Kleinebudde, 2020). For materials which were stated as brittle no minimum was detected (Antikainen and Yliruusi, 2003; Keizer and Kleinebudde, 2020). However, the observed trends for the pressure dependency of ER are heavily influenced by the accuracy of the measuring technique or the filtering and treatment of in-die force displacement curves. For microcrystalline cellulose the measured ER changes are up to 2.5 % (Keizer and Kleinebudde, 2020) or below 1.0 % (Antikainen and Yliruusi, 2003) in pressure ranges of 50–250 MPa. A statistical based experiment plan showed no significant influence of the pressure on the ER for microcrystalline cellulose in roll compaction (Luck et al., 2024).

### 1.4 Viscoelasticity and the kinetics of elastic recovery

Besides elastic, plastic and brittle fragmentation some materials show viscoelasticity (Picker, 2001; Rippie and Danielson, 1981; Sarkar et al., 2014). Viscoelasticity can be simplified described by a Kelvin-Voigt solid, where a spring and a dashpot are coupled in parallel (Rippie and Danielson, 1981). The elastic behaviour is therefore time dependent as the speed of ER is determined by the recovery of the dashpot. This leads to an exponential kinetics of the ER (Roeder, 2013). This was underlined be Sarkar et al. who investigated the kinetics of ER of tablets produced with different compression pressures after ejection (Sarkar et al., 2014). They emphasized that higher compression pressures led to less ER and a slower velocity of ER as well. This was contributed to the more plastically deformation behaviour of the used powder mixture containing different viscoelastic polymers as disintegrants. The same observation was made for tablets compressed at different dwell times containing viscoelastic materials like pregelatinized starch or microcrystalline cellulose (Haware et al., 2010). Longer dwell times reduced the overall amount and the velocity of ER. However, for a rather brittle material like dibasic calcium phosphate dihydrate no effect of the dwell time could be pointed out. In roll compaction the roll speed (RS) is correlated with the dwell time (Luck et al., 2022). Keizer and Kleinebudde showed that in uniaxial roll compaction simulation the mimicked RS had no influence on the in-die ER. Unrealistic high dwell times up to 3.0 s instead were proven to reduce the in-die ER (Keizer and Kleinebudde, 2020). The influence of the RS on the out-of-die ER and the kinetics of the ER was not investigated. Aim of this study is therefore to use a new established in-line elastic recovery measurement technique in roll compaction (Luck et al., 2024) to analyse the kinetics of ER and the effect of process parameters on ER.

## 2 Material and methods

### 2.1 Materials and raw material characterization

Pure Microcrystalline cellulose powder (MCC, Vivapur® 102, JRS Pharma, Germany) and two powder blends containing either MCC or fine powder hydroxypropyl cellulose (HPC, NISSO HPC SSL SFP, Nippon Soda, Japan) as binder and dibasic calcium phosphate anhydrous (DCPA, DI-CAFOS® A60, Chemische Fabrik Budenheim KG, Germany) as filler (Table 1) where evaluated regarding the ER at different positions along the roll (Section 2.2.). The detailed investigation of the ER kinetics was conducted using MCC (Pharmacel® 102, DFE Pharma, Germany) (Section 2.3.). Blending was performed with 30 rpm for 20 min using a lab-scale blender (LM40, L.B. Bohle Maschinen + Verfahren, Germany). For equilibration, all used materials were stored at 21 °C and 45 % relative humidity. The particle density ($\rho_{0}$) of the individual material was measured using AccuPyc 1330 helium pycnometer (Micromeritics, Norcross, USA) and the $\rho_{0}$ of the powder blends was estimated using the weighted harmonic mean of the individual $\rho_{0}$.

**Table 1:** Materials and formulations investigated in roll compaction.

Formulations	Proportion / %
MCC	100
MCC + DCPA	30 + 70
HPC + DCPA	10 + 90

### 2.2 In-line ribbon thickness measurement at different measurement positions

Roll compaction experiments were conducted using the MINI-PACTOR® (Gerteis Maschinen + Processengineering, Rapperswil-Jona, Switzerland) in gap-controlled mode equipped with smooth rolls (D=250mm and W=25mm) and rim roll sealing system. Process data was collected at a sample rate of 1 Hz. Full factorial designs of experiments (DoEs) with triplicated center point were utilized. Thereby, a triangulation laser LK-H087 (Keyence Deutschland, Neu-Isenburg, Germany) was used to measure the ${\mathrm{ER}}_{\mathrm{in}-\mathrm{line}}$ at a sample frequency of 50 Hz. The experimental method of the in-line ribbon thickness measurement using laser triangulation to calculate ${\mathrm{ER}}_{\mathrm{in}-\mathrm{line}}$ has been published in a previous study and was used as described (Luck et al., 2024). SCF, S and the roll RS were set as factors whereas ${\mathrm{ER}}_{\mathrm{in}-\mathrm{line}}$ and ${\mathrm{ER}}_{\mathrm{total}}$, the full elastic recovery after ribbon storage of 48 h, were investigated as responses. All factor levels are displayed in Table 2.

**Table 2:** Uncoded DoE factorial level at each measurement angle.

Experiments	SCF/ kN/cm	S/ mm	RS/ rpm
MCC	4.0–10.0	1.5–3.0	1.0–6.0
MCC + DCPA	7.0–13.0	2.0–3.0	2.0–6.0
HPC + DCPA	6.0–14.0	2.0–3.0	2.0–4.0

Each run was conducted for 5 min after reaching steady state conditions (ΔSCF±0.1kN/cm and ΔS±0.1mm) and the mean ribbon thickness in steady state ($∆\bar{x}$) can be calculated according to Eq. (1) with ${\bar{x}}_{\mathrm{empty}}$ as the mean distance to the roll without ribbon attached and ${\bar{x}}_{\mathrm{ribbon}}$ as mean distance to the roll with ribbon attached. $∆\bar{x}$ can be used to determine ${\mathrm{ER}}_{\mathrm{in}-\mathrm{line}}$ according to Eq. (2).

(1) $$∆\bar{x}={\bar{x}}_{\mathrm{empty}}-{\bar{x}}_{\mathrm{ribbon}}$$

(2) $${\mathrm{ER}}_{\mathrm{in}-\mathrm{line}}=\left[\frac{\left(∆\bar{x}-S\right)}{S}\right]*100\%$$

**Fig. 1:**
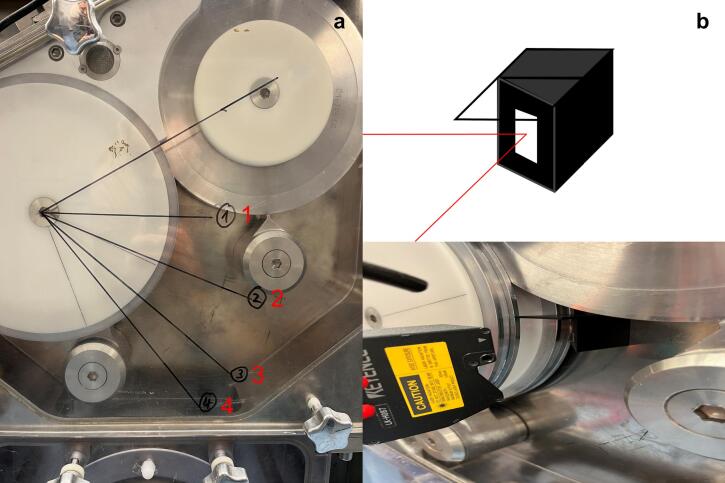
Representation of the four established measurement positions for the in-line laser triangulation measurement along the master roll (**a**). Usage of mirrors (white) and 3D printed mirror holder (black) to enable measurement position 1 (**b**).

In this study, ${\mathrm{ER}}_{\mathrm{in}-\mathrm{line}}$ was determined at different angles (β) of 30°, 50°, 70° and 85° referred to the gap named as position 1, 2, 3 and 4 in Fig. 1a. In total 12 DoEs, three formulations (Table 1) at four measurement angles (Fig. 1a), with 11 runs each were utilized. At any measurement the laser aimed at the centre of the master roll in the middle of W. As the size of the used laser system and the measuring range was not compatible with position 1, two 10 × 10 mm silver coated flat surface mirrors (Edmund Optics GmbH, Mainz, Germany) were attached to a 3D printed holder and placed near position 1 (Fig. 1b). The mirrors are specified as the coating reflects 98 % of the light with a wavelength of above 450 nm on average. The red laser with a wavelength of 655 nm aimed at 45° on the mirror surface and the reflection targets at the middle of the roll width (Fig. 1b). This enables to gain data as close as possible to the gap.

By division of β with the angular velocity (ω) in °/s of the rolls (Eq. (3)) the time (t) at each measurement position, after the powder passed S, can be calculated. ω can be determined by referring the RS per second to 360° of a full roll rotation (Eq. (4)).

(3) t=β/ω

(4) $$\omega =360^{{}^{\circ}}\times \mathrm{RS}$$

### 2.3 Ribbon thickness measurement after process stop

To analyse the kinetics of the ER as MCC ribbon relaxation over time, the change of $x_{\mathrm{ribbon}}$ over time (${\mathrm{\Delta x}}_{\mathrm{ribbon}}$) was determined. The previous established experimental design was changed slightly and a 2^2^-factorial design with three center point runs (11 runs in total) was performed keeping S constant at 3.0 mm. The roll compactor was stopped after 3 min in steady state and the ${\mathrm{\Delta x}}_{\mathrm{ribbon}}$ was measured over a 5-min period of time without roll rotation. The identical equipment was used as described previously and the triangulation laser measurement was performed only at measurement position 2 with 50° to the gap (Fig. 1a).

**Fig. 2:**
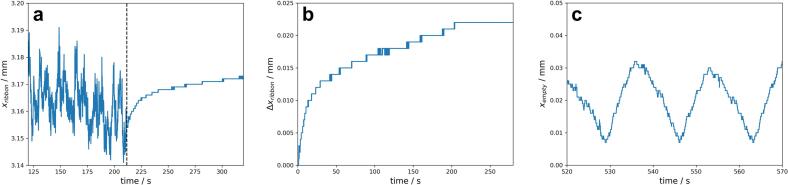
Raw data of the $x_{\mathrm{ribbon}}$ measurement of MCC with 7 kN/cm, 3.0 mm and 3.5 rpm using laser triangulation; vertical line marks $t_{\mathrm{start}}$ (**a**) and the corresponding ${\mathrm{ER}}_{\mathrm{in}-\mathrm{line}}$ kinetic with $t_{\mathrm{start}}$ set to zero (**b**). **c** displays $x_{\mathrm{empty}}$ after the ribbon were scratched off. All plots show the same individual run.

As the triangulation laser measures over the entire process a starting point ($t_{\mathrm{start}}$) in the measurement data has to be defined manually to analyse the kinetics of the ${\mathrm{ER}}_{\mathrm{in}-\mathrm{line}}$. $t_{\mathrm{start}}$ was set manually as close as possible after the typical scattering of $x_{\mathrm{ribbon}}$ has been stopped (vertical line in Fig. 2a). An example of $x_{\mathrm{ribbon}}$ measured over time is shown in Fig. 2a. After the fluctuations had stopped, due to the stop of the roll compactor the kinetics of ${\mathrm{ER}}_{\mathrm{in}-\mathrm{line}}$ can be investigated. To make the observed kineticss comparable, $t_{\mathrm{start}}\;$was set to zero (Fig. 2b) and ${\mathrm{\Delta x}}_{\mathrm{ribbon}}$ was plotted as a function of time. The end point was set as $t_{\mathrm{start}}+280\;s$. Fig. 2c shows the fluctuation of $x_{\mathrm{empty}}$ measured after the ribbon was scratched off the roll.

The processed data (Fig. 2b) were fitted using the curve fit tool of SciPy (Version 1.10.1.) of Python (Version 3.9.18.) with the environment of Jupyter Notebook (Version 6.5.4.). The fit was utilized using Eq. (5). The optimal parameters for a, b and k were determined, gave one exponential fit and R^2^ was investigated using scikit-learn (Version 1.3.0.).

(5) $$f\left(t\right)=a*\left(b-e^{-k*t}\right)$$

To optimize the fit of the kinetics curve two exponential fits, named ${\mathrm{ER}}_{A}$ and ${\mathrm{ER}}_{B}$*,* were combined. Both of the form shown in Eq. (5). The separation and thus the starting point of ${\mathrm{ER}}_{B}$ was set as the first determined intersection of the one exponential fit with the raw data (example in Fig. 6a and c). R^2^ was contained as described above for both exponential fits. A sigma minus plot was carried out to analyse the kinetics of ${\mathrm{ER}}_{B}$*.* Therefore, $\ln\left(\lim_{t\to \infty }\left({\mathrm{\Delta x}}_{\mathrm{ribbon}}\right)-{\mathrm{\Delta x}}_{\mathrm{ribbon}}\left(\mathrm{time}\right)\right)$ was plotted against time. Slope (m) and the coefficient of determination R^2^ of the linear sigma minus plot were given. A sensitivity analysis was carried out to test the influence of a change in the $t_{\mathrm{start}}$ determination or m. In relation to this $t_{\mathrm{start}}$ was shifted by 1.0, 2.0 and 5.0 s for all three centre point runs. The extrapolated maximum ER which can be measured in-line (${\mathrm{ER}}_{\mathrm{in}-\mathrm{line}\_\max}$) can be calculated using Eq. (6). ${\mathrm{ER}}_{\mathrm{in}-\mathrm{line}\_\max}$ was compared to ${\mathrm{ER}}_{\mathrm{total}}$.

(6) $${\mathrm{ER}}_{\mathrm{in}-\mathrm{line}\_\max}=\left[\frac{\left(\left(∆\bar{x}+\lim_{t\to \infty }\left({\mathrm{\Delta x}}_{\mathrm{ribbon}}\right)\right)-S\right)}{S}\right]*100$$

### 2.4 Ribbon characterization

#### 2.4.1 Estimation of ${\mathrm{SF}}_{\mathrm{gap}}$

According to previously published literature (Luck et al., 2024; Sousa et al., 2020) the dimensionless Midoux number can be used to estimate the ribbon density at gap width ($\rho_{\mathrm{Mi}}$). With regard to this, Eq. (7) was applied to calculate $P_{\max}$. $P_{\max}$ can be utilized to get $\rho_{\mathrm{Mi}}$ using the linear regression between $\ln\left(\mathrm{tableting pressure}\right)$ and $\ln\left(\mathrm{tablet density}\right)$ out of single punch compression experiments (Luck et al., 2022). By referring $\rho_{\mathrm{Mi}}$ to the particle density of the powder ($\rho_{0}$) ${\mathrm{SF}}_{\mathrm{Mi}}$, the solid fraction at gap width, can be predicted (Eq. (8)). As ${\mathrm{SF}}_{\mathrm{Mi}}$ seems to be a suitable indicator for ${\mathrm{SF}}_{\mathrm{gap}}$ (Sousa et al., 2020), ${\mathrm{SF}}_{\mathrm{Mi}}$ and ${\mathrm{SF}}_{\mathrm{gap}}$ were used equivalent in this study.

(7) $$P_{\max}=\frac{2\mathrm{SCF}}{D}\times \sqrt{\frac{2K}{\mathrm{\pi S}/D}}$$

(8) $${\mathrm{SF}}_{\mathrm{Mi}}=\frac{\rho_{\mathrm{Mi}}}{\rho_{0}}$$

#### 2.4.2 Determination of ${\mathrm{SF}}_{\text{ribbon}}$

Powder pycnometry measurements of the ribbons were done using the GeoPyc 1360 powder pycnometer (Micromeritics, Norcross, USA). Experimental implementation was done according to previously published standardized procedure (Luck et al., 2022). However, individual measurements instead of triplicates were performed. Eq. (9) gives the ribbon solid fraction (${\mathrm{SF}}_{\mathrm{ribbon}})\;$after elastic recovery with minimum of 48 h storage after production.

(9) $${\mathrm{SF}}_{\mathrm{ribbon}}=\frac{\rho_{\mathrm{ribbon}}}{\rho_{0}}$$

#### 2.4.3 Determination of ${\mathrm{ER}}_{\text{total}}$

The full ER after minimum 48 h of ribbon production can be gained following Eq. (10) (Yohannes et al., 2015). To proof the results of Eq. (10) the ribbon thickness after full ER (x) was measured in the middle of the ribbon width using a calliper (Absolute AOS Digimatic, Mitutoyo, Kawasaki, Japan) and the ${\mathrm{ER}}_{\mathrm{total}}$ was calculated according to Eq. (11). Therefore, MCC ribbons were produced at increasing SCF of 4–12 kN/cm, constant S of 2.0 mm and RS of 2.0 rpm.

(10) $${\mathrm{ER}}_{\mathrm{total}}=\left[\frac{\left(\;{\mathrm{SF}}_{\mathrm{gap}}-{\mathrm{SF}}_{ri\mathrm{bbon}}\right)}{{\mathrm{SF}}_{\mathrm{ribbon}}}\right]*100$$

(11) $${\mathrm{ER}}_{\mathrm{total}}=\left[\frac{\left(x-S\right)}{S}\right]*100\%$$

#### 2.4.4 XμCT imaging of ribbons

Ribbons of pure MCC were imaged using the CT-ALPHA (ProCon X-ray, Sarstedt, Germany) to analyse whether lamination or splitting could potentially distort the measurement of ER. The voltage was set to 80 kV and the amperage to 50 μA. 1600 images with a voxel size of 15 μm per rotation were taken. The software VGStudio 3.0.1. (Volume Graphics GmbH, Heidelberg, Germany) was used for the reconstruction of the raw images.

## 3 Results and discussion

### 3.1 Comparison of ${\mathrm{ER}}_{\mathrm{total}}$ determination methods

**Fig. 3:**
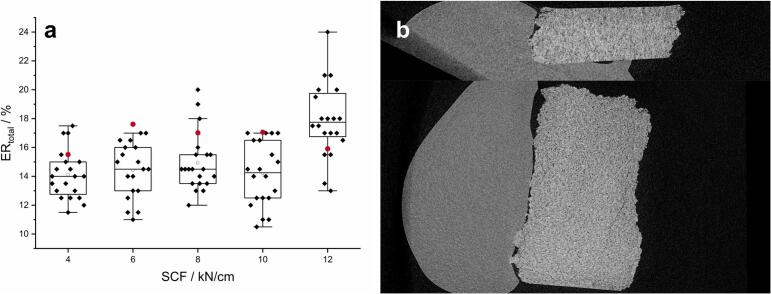
Comparison of ${\mathrm{ER}}_{\mathrm{total}}$ measurement (**a,** boxplots, black data points) and ${\mathrm{ER}}_{\mathrm{total}}$ calculations using the SF (**a**, red data points). Boxes are displayed with interquartile range (IQR), individual data points (♦), mean (□), median (—) and 1.5 IQR (errorbar). Vertical (**b**, upper) and horizontal (**b**, lower) XμCT cross section of a MCC ribbon (light grey). The ribbon holder is shown in dark grey. (For interpretation of the references to colour in this figure legend, the reader is referred to the web version of this article.)

The measurement of the ribbon thickness of MCC ribbons using a calliper (Section 2.4.3.) led to ${\mathrm{ER}}_{\mathrm{total}}$ values of 10 to 20 % with SCF of 4–10 kN/cm and a S of 2.0 mm. For 12 kN/cm a tendency to higher ${\mathrm{ER}}_{\mathrm{total}}$ could be detected (Fig. 3a, black). Except of one case the calculated ${\mathrm{ER}}_{\mathrm{total}}$ based on ${\mathrm{SF}}_{\mathrm{ribbon}}$ and ${\mathrm{SF}}_{\mathrm{gap}}$ (Eq. (10) and Fig. 3a, red) was within the 1.5 IQR and could not be detected as outlier of the data set. As ${\mathrm{SF}}_{\mathrm{Mi}}$ is a model based estimator for ${\mathrm{SF}}_{\mathrm{gap}}$, deviations of measured and calculated ${\mathrm{ER}}_{\mathrm{total}}$ values might be explainable due to the offset of the model itself. However, both ${\mathrm{ER}}_{\mathrm{total}}$ determination methods led to comparable results and are able to represent the ER of ribbons. ${\mathrm{ER}}_{\mathrm{total}}$ was calculated and compared with ${\mathrm{ER}}_{\mathrm{in}-\mathrm{line}\_\max}$ (Section 2.3.). XμCT cross section images showed no or only minor cracks and no splitting or lamination could be observed which might had influence on the measurement of ER (Fig. 3b).

### 3.2 Kinetics of ER after process stop

To analyse the ${\mathrm{ER}}_{\text{in}-\text{line}}$ two different approaches were carried out. The first is presented in Section 3.2. included the measurement of the ribbon thickness of pure MCC ribbons over time at $\beta =50^{{}^{\circ}}$ after the process has been stopped. The second approach deals with the measurement of ${\mathrm{ER}}_{\text{in}-\text{line}}$ at different measurement positions (Fig. 1a) using three different formulations (Section 3.3.).

**Fig. 4:**
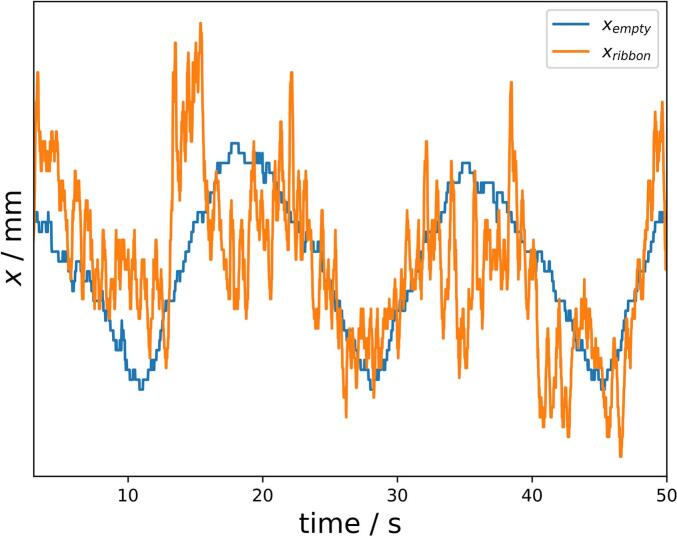
Overlay of unprocessed $x_{\mathrm{empty}}$ (Fig. 2c) and $x_{\mathrm{ribbon}}$ (Fig. 2a, 150–200 s) of the same center point run with the RS of 3.5 rpm.

In the first approximately 215 s a typical pattern of $x_{\mathrm{ribbon}}$ fluctuations was visible (Fig. 2a; Fig. 4, orange). This fluctuation pattern can be explained by a small imbalance of the rotating master roll which can be measured as fluctuation of $x_{\mathrm{empty}}$ (Fig. 2c; Fig. 4, blue). The fluctuation pattern matches quite well the frequency of one roll rotation and correlates with the RS of 3.5 rpm in the shown example (Fig. 4). However, the span $x_{\mathrm{ribbon}\_\max}-x_{\mathrm{ribbon}\_\min}$ of 0.044 mm was greater then $x_{\mathrm{empty}\_\max}-x_{\mathrm{empty}\_\min}$ of 0.025 mm. The fluctuation of the measured ribbon thickness can therefore be attributed to the imbalance of the roll itself and probably also to the fluctuation of the S over the test period. The majority of the fluctuation was caused by the roll imbalance.

**Fig. 5:**
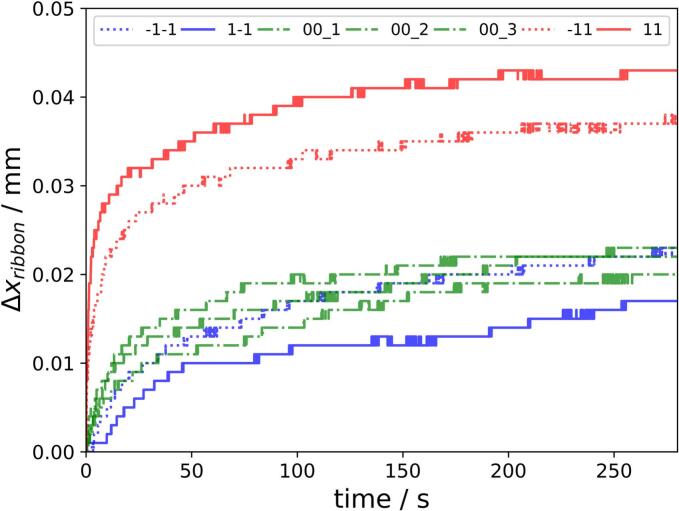
Kinetic of the ${\mathrm{ER}}_{\mathrm{in}-\mathrm{line}}$ with ${\mathrm{\Delta x}}_{\mathrm{ribbon}}$ at low = −1 (blue), medium = 0 (green) and high =1 (red) RS with low = −1 (dotted), medium = 0 (alternating) and high = 1 (solid) SCF. Individual experiments displayed. (For interpretation of the references to colour in this figure legend, the reader is referred to the web version of this article.)

In the following, Fig. 5 gives an overlook of the measured ER after the process had been stopped including all 7 Runs of the utilized DoE (Section 2.3.). The ER is expressed as ${\mathrm{\Delta x}}_{\mathrm{ribbon}}$ at different SCF and RS combinations. The ER kinetics can be described as exponential kinetics in accordance to the literature (Keizer and Kleinebudde, 2020; Sarkar et al., 2014). The stepwise increase of ${\mathrm{\Delta x}}_{\mathrm{ribbon}}$ is due to the resolution limit of the used triangulation laser. But it has to be mentioned that the majority of ER took place before the ribbon even reaches the measurement spot. ${\mathrm{ER}}_{\mathrm{in}-\mathrm{line}\_\max}$ was 5.75±0.89%, expressed as mean ± standard deviation, which consists of 4.63±1.01% ${\mathrm{ER}}_{\mathrm{in}-\mathrm{line}}$ before $t_{\mathrm{start}}$ and only 1.12±0.23% of the ER can be measured after $t_{\mathrm{start}}$ (Fig. 2a and 5). In comparison to ${\mathrm{ER}}_{\mathrm{in}-\mathrm{line}\_\max}$, ${\mathrm{ER}}_{\mathrm{total}}$ with 23.64±1.72% was approximately fourfold higher. Hereby ${\mathrm{ER}}_{\mathrm{in}-\mathrm{line}}$ and ${\mathrm{ER}}_{\mathrm{total}}$ values of MCC are in good agreement with previous published literature (Luck et al., 2024). This emphasized that the full ER is not possible if the ribbon is stuck on the roll surface and trapped between both sealing rims. The largest part of the ER has to take place after the ribbons are scratched off the roll. The same observation was published by Train who investigated ER in the direct compression of powders (Train, 1956). He stated that ER took place in only limited way in the tableting die. Further ER was only possible after ejection of the compact out-of-die due to the friction in-die.

However, the measurable part of the ER can be used to investigate the kinetics. It can be divided in an ${\mathrm{ER}}_{A}$ which is characterized by a much higher slope than the following ${\mathrm{ER}}_{B}$ (Fig. 5). Same trends of the kinetics were observed by measuring the ER of tablets containing MCC, Hydroxypropyl Methylcellulose, Cellulose Acetate and Carrageenan using a micrometer screw (Picker, 2001). The tablet height had risen sharply immediately after ejection of the tablets. The kinetics then showed saturation.

The importance of the ${\mathrm{ER}}_{A}$ is higher with increasing RS (Fig. 5, red) leading to a higher rise of the ER at the beginning of the curves. Thus, shorter compaction time leads to higher ${\mathrm{ER}}_{A}$ due to the shorter time for plastic deformation of the used MCC. This was supported by the results of Muthancheri et al. who describe the kinetics of compaction as dwell time dependent for plastic/viscoelastic materials (Muthancheri et al., 2024). This implies a dwell time dependency for the corresponding ER as well. However, the dwell time is not solely determined by the RS. The roll diameter determines the nip angle (Kleinebudde, 2022) and the therefore the dwell time which might has an influence on the ER using viscoelastic materials as binder (Diener et al., 2022). In fact smaller roll diameter would lead to an extended dwell time which might reduce ER.

An effect of the SCF on the ${\mathrm{ER}}_{A}$ was not visible. Higher RS seems to cause a higher overall ER (Fig. 5), which was supported by Katz et al. who showed that a higher tip speed resulted in a higher correction factor to align the predicted solid fraction based on in-die data to the measured out-of-die solid fraction of tablets (Katz et al., 2013). This was referred to the viscoelastic properties of pregelatinized starch. It has to be mentioned that $t_{\mathrm{start}}$ is different for different RS but it had no influence on the interpretation of the results as ${\mathrm{ER}}_{\mathrm{in}-\mathrm{line}}$, meaning the starting condition of the measurement, is comparable for all RS at the same measurement position (Fig. 9a).

**Fig. 6:**
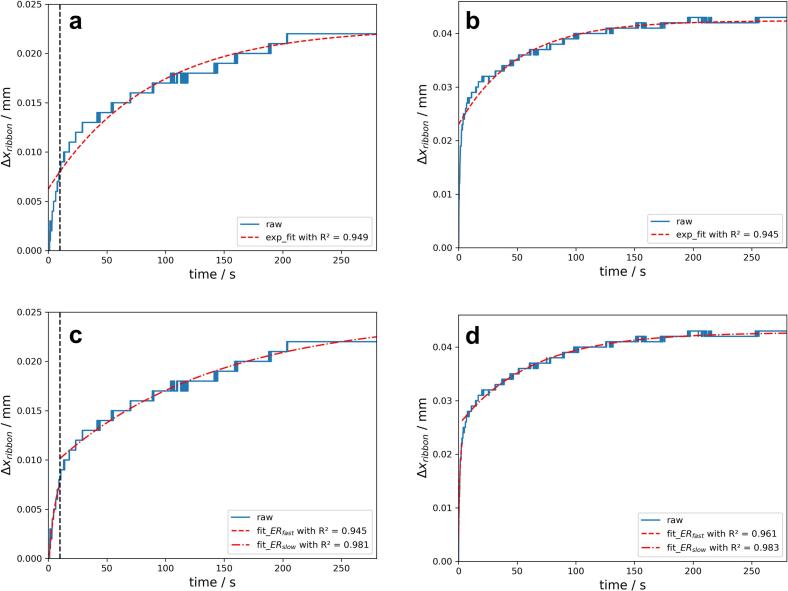
Example of one exponential fit (dashed red line in **a** and **b**) and two exponential fit of ${\mathrm{ER}}_{A}$ (dashed red line) and ${\mathrm{ER}}_{B}$ (alternating red line) (**c** and **d**) of the kinetic of ${\mathrm{ER}}_{\mathrm{in}-\mathrm{line}}$. **a** and **c** refers to a centre point (7.0 kN/cm, 3.5 rpm) and **b** and **d** to a high SCF and RS (10.0 kN/cm, 6.0 rpm). The vertical dashed black line in **a** and **c** gives an example of the starting point of ${\mathrm{ER}}_{B}$. Individual runs plotted. (For interpretation of the references to colour in this figure legend, the reader is referred to the web version of this article.)

Fig. 6a and b show two examples of the one exponential fit for ${\mathrm{ER}}_{\mathrm{in}-\mathrm{line}}$ following Eq. (5). With one exponential function no sufficient fit of the represented data can be reached as the fit is more suitable for ${\mathrm{ER}}_{B}$ but ${\mathrm{ER}}_{A}$ cannot be illustrated.

Therefore, two exponential fits were carried out to fit the ER kinetics more sufficiently (Fig. 6c and d). In the shown example R^2^ of ${\mathrm{ER}}_{A}$ and ${\mathrm{ER}}_{B}$ are minimum 0.945 and the kinetics of ${\mathrm{ER}}_{\mathrm{in}-\mathrm{line}}$ is in good agreement with the displayed fits. Except one run the better fit can be reached for ${\mathrm{ER}}_{B}$ as R^2^ is higher than for ${\mathrm{ER}}_{A}$ (Table 3). As R^2^ of ${\mathrm{ER}}_{B}$ are in ranges of 0.925–0.987 robust fits could be observed which enables further evaluation of ${\mathrm{ER}}_{B}$ (Section 2.3).

**Table 3:** Goodness of fit represented as R^2^ and parameters a, k for ${\mathrm{ER}}_{A}$ and ${\mathrm{ER}}_{B}$ using MCC. Each run of the DoE is designated as an uncoded factor level combination, e.g. 4.0 kN/cm + 1.0 rpm.

Run	Kinetics	a	k	R^2^
4.0 kN/cm + 1.0 rpm	${\mathrm{ER}}_{A}$	0.013	0.079	0.911
	${\mathrm{ER}}_{B}$	0.018	0.010	0.987
10.0 kN/cm + 1.0 rpm	${\mathrm{ER}}_{A}$	0.018	0.065	0.966
	${\mathrm{ER}}_{B}$	0.015	0.011	0.925
7.0 kN/cm + 3.5 rpm	${\mathrm{ER}}_{A}$	0.012/	0.146/	0.945/
		0.007/	0.206/	0.545/
		0.008	0.193	0.960
	${\mathrm{ER}}_{B}$	0.015/	0.007/	0.981/
		0.016/	0.008/	0.983/
		0.014	0.013	0.976
4.0 kN/cm + 6.0 rpm	${\mathrm{ER}}_{A}$	0.015	0.461	0.951
	${\mathrm{ER}}_{B}$	0.016	0.014	0.970
10.0 kN/cm + 6.0 rpm	${\mathrm{ER}}_{A}$	0.019	0.849	0.961
	${\mathrm{ER}}_{B}$	0.017	0.016	0.983

**Fig. 7:**
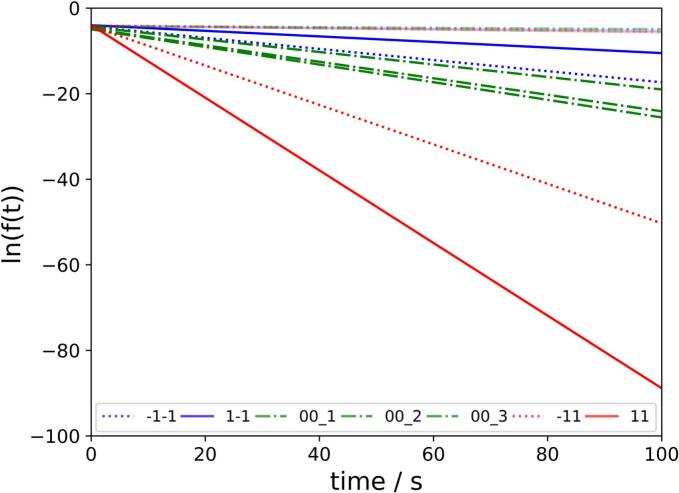
Linearised form of ${\mathrm{ER}}_{A}$ and ${\mathrm{ER}}_{B}$(faded colours) with slope −k and y axis intercept $\ln\left(a\right)$ at low = −1 (blue), medium = °0 (green) and high = °1 (red) RS with low = −1 (dotted), medium = 0°(alternating) and high = 1 (solid) SCF. Individual experiments displayed. (For interpretation of the references to colour in this figure legend, the reader is referred to the web version of this article.)

${\mathrm{ER}}_{A}$ is characterized by a higher rise of the curve compared to ${\mathrm{ER}}_{B}$ which correlated with higher k values (Table 3). However, a seems to be similar and therefore ln(a) as y axis intercept after linearisation (Eq. (12)) is quiet similar as well (Fig. 7).

(12) $$\ln\left(f\left(t\right)\right)=\ln\left(a\right)-\mathrm{kt}$$

In addition higher RS led to higher k values and slopes for ${\mathrm{ER}}_{A}$ (Fig. 7). But the RS showed no obvious effect on k for ${\mathrm{ER}}_{B}$ and therefore were shown in faded colours (Fig. 7). In the case of ${\mathrm{ER}}_{B}$, k only increases slightly with increasing SCF, while for ${\mathrm{ER}}_{A}$, k increases significantly, meaning that the difference becomes greater with increasing SCF and RS (Table 3, Fig. 7). The three center point runs at 7.0 kN/cm and 3.5 rpm showed comparable results for k of ${\mathrm{ER}}_{A}$ (Fig. 7) and ${\mathrm{ER}}_{B}$ (Table 3).

**Fig. 8:**
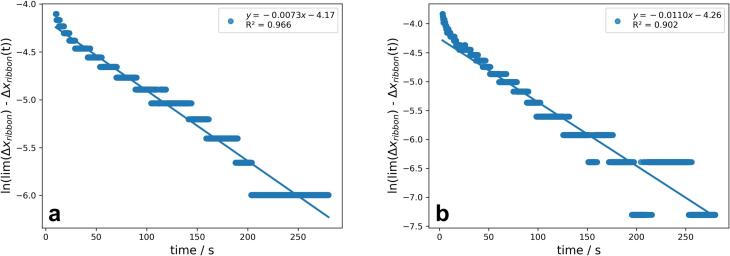
Sigma minus plot of ${\mathrm{ER}}_{B}$ for one centre point (**a**) and high SCF and RS (**b**). Linear regression with function and R^2^ of individual runs displayed.

Sigma minus plot analysis was utilized to further analyse ${\mathrm{ER}}_{B}$ (Fig. 8). In all cases linear regressions with R^2^ of higher 0.902 could be observed. All values of R^2^ and the corresponding slope of the sigma minus regression m are shown in Table 4.

**Table 4:** Slope m and R^2^ for the sigma minus plot of ${\mathrm{ER}}_{B}$. Runs of the DoE are named with the uncoded factor level combinations.

Run	m	R^2^
4.0 kN/cm + 1.0 rpm	−0.0052	0.973
10.0 kN/cm + 1.0 rpm	−0.0055	0.923
7.0 kN/cm + 3.5 rpm	−0.0073 / -0.0084 / -0.0075	0.966 / 0.970 / 0.954
4.0 kN/cm + 6.0 rpm	−0.0097	0.941
10.0 kN/cm + 6.0 rpm	−0.0110	0.902

**Fig. 9:**
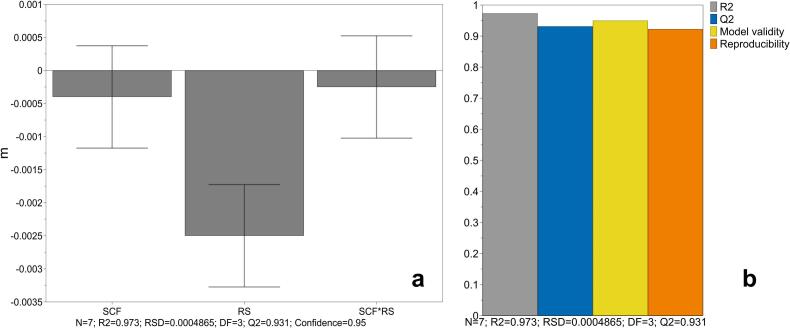
Coefficient plot (**a**) and Summary of Fit (**b**) for the model to investigate the effect of SCF, RS and the interaction of SCF and RS on m. Mean coefficient ± 95 % confidence interval.

Higher RS leads to higher slope for the sigma minus plot of ${\mathrm{ER}}_{B}$ (Fig. 9a) which means that ${\mathrm{ER}}_{B}$ is accelerated and the exponential function strives more quickly towards $\lim_{t\to \infty }\left({\mathrm{\Delta x}}_{\mathrm{ribbon}}\right)$. Therefore, the ER is faster. This observation is comparable to the conclusion of Section 3.2. where ${\mathrm{ER}}_{A}\;$was also more pronounced at higher RS. SCF and interaction of SCF and RS have no significant effect on m (Fig. 9a). Overall, the model showed a good reproducibility over 0.9 (Fig. 9b).

The stepwise increase of the ${\mathrm{\Delta x}}_{\mathrm{ribbon}}$ (Fig. 6) is also illustrated in Fig. 8 as stepwise decrease of $\ln\left(\lim_{t\to \infty }\left({\mathrm{\Delta x}}_{\mathrm{ribbon}}\right)-{\mathrm{\Delta x}}_{\mathrm{ribbon}}\left(t\right)\right)$. As the ER can be only successful described with two different exponential kineticss, ${\mathrm{ER}}_{A}$ and ${\mathrm{ER}}_{B},$ the linear fit is not always sufficient at the transition of ${\mathrm{ER}}_{A}$ and ${\mathrm{ER}}_{B}$ (compare Fig. 8a and 8b) and hardly dependent on the determination of the $t_{\mathrm{start}}$ of ${\mathrm{ER}}_{B}$.

To detect the possible effect of a change in $t_{\mathrm{start}}$ on m a sensitivity analysis was performed. A maximum change of −0.0005 for m could be observed when $t_{\mathrm{start}}$ was shifted 5 s. Thus, m seems to be less sensitive towards change in $t_{\mathrm{start}}$. The change is approximately fourfold smaller than the difference in m caused by the RS factor level adaption.

### 3.3 ${\mathrm{ER}}_{\text{in}-\text{line}}$ at different measurement positions

The time, t at the measurement positions after the ribbon were compacted, differs with changing RS (settings).

**Fig. 10:**
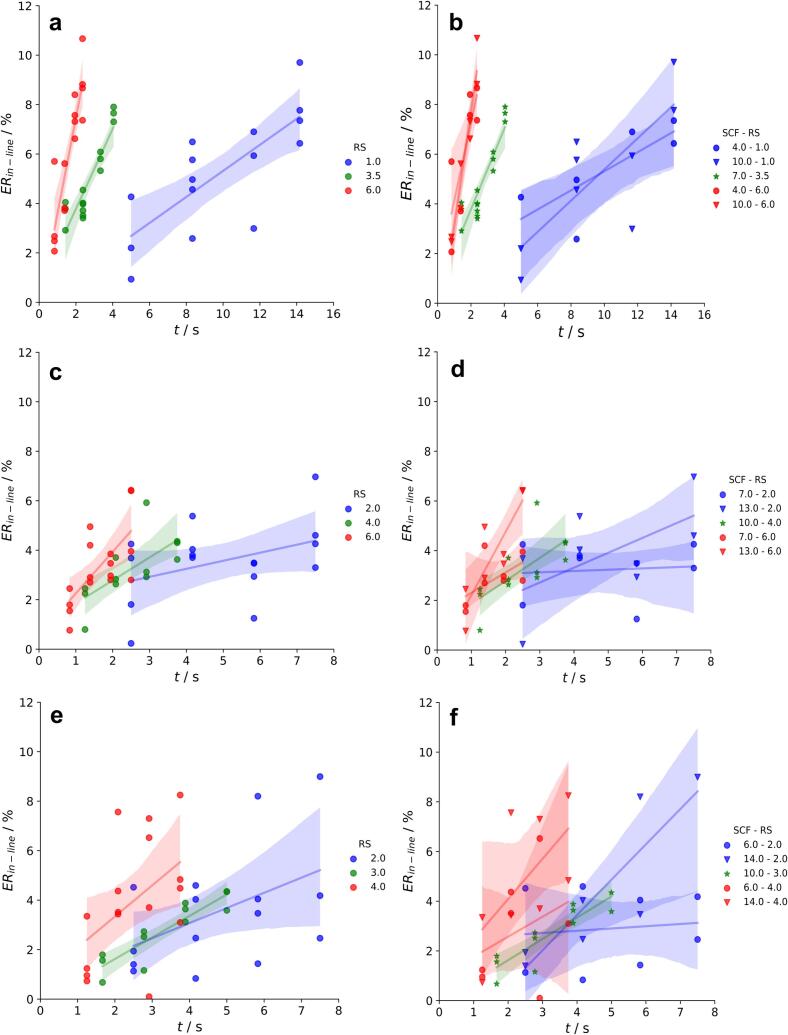
${\mathrm{ER}}_{\mathrm{in}-\mathrm{line}}$ of MCC (**a** and **b**), MCC + DCPA (**c** and **d**) and HPC + DCPA (**e** and **f**) ribbons in dependence of t with different RS (**a**, **c** and **e**) and SCF−RS (**b**, **d** and **f**) settings. Each data point represents the result of one run in the DoE. Solid lines illustrate linear regressions with 95 % confidence intervals.

For example, with 1.0 rpm the ribbon reaches the last measurement point ($\beta =85^{{}^{\circ}}$) in about 14.2 s. With sixfold higher RS of 6.0 rpm $\beta =85^{{}^{\circ}}$ is passed in only 2.4 s (Fig. 10a and b). To calculate ${\mathrm{ER}}_{\text{in}-\text{line}}$ at each measurement position, $∆\bar{x}$ was measured and referred to the gap-width S (Eq. (2)). As example of the results at 70° all conducted process data (SCF,S,RS) and the measured $∆\bar{x}$, ${\mathrm{SF}}_{\mathrm{Mi}}$ and ${\mathrm{SF}}_{\mathrm{ribbon}}$ for each formulation are shown in tables S1-S3 in the supplemental material. The determined ${\mathrm{ER}}_{\text{in}-\text{line}}$ increased with increasing t for all RS and formulations (Fig. 10a, c and e). However, higher RS changed the slope of the regression between t and ${\mathrm{ER}}_{\text{in}-\text{line}}$. Higher RS results in faster increase of ${\mathrm{ER}}_{\text{in}-\text{line}}.$ The kinetics which seems to be linear is in fact not as it is shown in Section 3.2. The measurements shown here represent the pseudo linear ER. To underline the results of Section 3.2. the slope of ER was also increased at higher RS. A change in SCF had no effect on the velocity of ER for pure MCC (Fig. 10b).

To investigate the effect of an addition of brittle materials, powder blends with DCPA were analysed. The results are illustrated in Fig. 10c to 10f. The velocity of the ${\mathrm{ER}}_{\text{in}-\text{line}}$ is again dependent on the RS (Fig. 10c and e). Overall, the more brittle and less elastic characteristic of the MCC + DCPA blend led to slopes of 0.3, 0.9 and 1.6 %/s with increasing RS. In comparison the more viscoelastic pure MCC showed slopes of 0.5, 1.6 and 3.9 %/s. Even the slightly higher RS used for the powder blend did not overcome this effect. Thus, introduction of brittle materials reduced the effect of RS on the ER kinetics. This can be allied with the results of Li et al. how observed a decreasing time dependency in compaction of MCC blends with increasing lactose content and put the effect of the roll speed in context with ER, which is influenced by the material properties (Li et al., 2024).

**Fig. 11:**
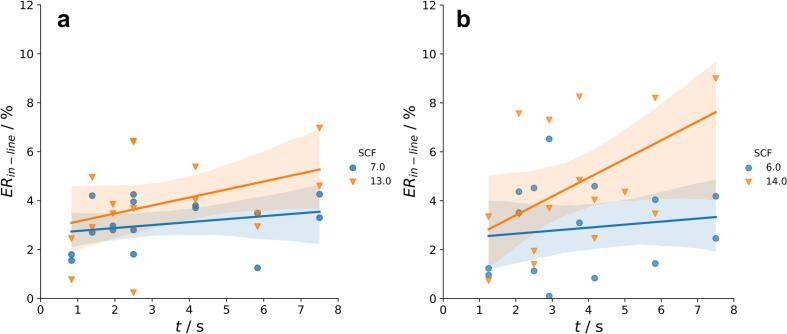
${\mathrm{ER}}_{\mathrm{in}-\mathrm{line}}$ of MCC + DCPA (**a**) and HPC + DCPA (**b**) ribbons in dependence of t with different SCF settings. Each data point represents the result of one run in the DoE. Solid lines illustrate linear regressions with 95 % confidence intervals.

Moreover it is supported by Haware et al. who could not detect an effect of the dwell time on the ER of tablets containing only brittle material like lactose (Haware et al., 2010). However, the used powder blend still contains MCC which explains the visible effect of the RS on the ER kinetics. Comparing Fig. 10a and c/10e the fluctuations and the confidence intervals are much broader for the powder blend. This can be explained by the effect of the SCF which is not visible for pure MCC (Fig. 10b and d). Same trends are visible for the blend of HPC + DCPA (Fig. 10e and f). With 13.0/14.0 kN/cm, ${\mathrm{ER}}_{\text{in}-\text{line}}$ values are overall higher than with 6.0/7.0 kN/cm (Fig. 11a and b). If the brittle DCPA is added to a more plastically behaving binder like MCC or HPC, SCF seems to become important for the ER. The increase of the ER with higher pressures is well known for brittle materials (Mahmah et al., 2019) and is also detectable in binary mixtures (Hirschberg et al., 2020). An equal phenomena was published by Diener et al. who established an direct-gap detection measurement system to investigate the ER of Lithium-Ion Battery Cathodes in roll compaction. It was emphasized that the ER increased with higher SCF in a formulation with only a low plastic binder content (Diener et al., 2022).

## 4 Conclusion

This study describes the in-line determination of the kinetics of ribbon ER in roll compaction. It was emphasized that ${\mathrm{ER}}_{\mathrm{in}-\mathrm{line}}$ consists of two exponential kineticss with different behaviour. However, the majority of the ER available on the roll surface (4.63±1.01%) took place before the desired measurement spot was reached by the ribbon and could therefore not be detected by this method. The measurable part of the ER (1.12±0.23%) showed characteristics of a ${\mathrm{ER}}_{A}$ which consisted a higher rise, followed by a flattening kinetics ${\mathrm{ER}}_{B}$. Overall values of ${\mathrm{ER}}_{\mathrm{in}-\mathrm{line}\_\max}$ are fourfold lower than the full ER of the ribbons, ${\mathrm{ER}}_{\mathrm{total}}$ (23.64±1.72%). This indicates that complete ER is suppressed by the sticking of the ribbon on the roll and between the sealing rims. The main part of the ER (≈23.6%−4.6%−1.1%≈17.9%) took place after the ribbons were scratched off. For viscoelastic materials like MCC, RS has an effect on both ${\mathrm{ER}}_{A}$ and ${\mathrm{ER}}_{B}$. Higher RS and therefore lower dwell time under compaction results in faster ER due to the shorter time for plastic deformation. The elastic deformation predominates. The addition of a brittle material to the blend reduces the effect of the RS on the velocity of ER but is still visible. On the other hand, the SCF becomes important if brittle DCPA was compacted and higher SCF leads to an increase in ER. The conducted study shows a novel approach to characterize the ER kinetics and behaviour of materials and blends used for roll compaction. This study supports the understanding and characterization of relaxation times and the effect of the RS and SCF in roll compaction.

## CRediT authorship contribution statement

**Martin Lück:** Visualization, Methodology, Investigation, Conceptualization. **Stefan Klinken-Uth:** Writing – review & editing, Methodology. **Peter Kleinebudde:** Writing – review & editing, Supervision, Conceptualization.

## Declaration of competing interest

PK is member of the EAB of Int J Pharm X. If there are other authors, they declare that they have no known competing financial interests or personal relationships that could have appeared to influence the work reported in this paper.

## Data Availability

The datasets generated during and/or analysed during the current study are available from the corresponding author on reasonable request.

## Appendix A Supplementary data

Table S1: MCC: Process parameters (SCF, S and RS) with the measured ribbon thickness (∆*x*), the estimated solid fraction of the ribbons at-gap (*SF*) and solid fraction of the ribbons after elastic recovery (*SF*) in randomized order as conducted at the measurement angle of 70°. Table S2: MCC+DCPA: Process parameters (SCF, S and RS) with the measured ribbon thickness (∆*x*), the estimated solid fraction of the ribbons at-gap (*SF*) and solid fraction of the ribbons after elastic recovery (*SF*) in randomized order as conducted at the measurement angle of 70°. Table S3: HPC+DCPA: Process parameters (SCF, S and RS) with the measured ribbon thickness (∆*x*), the estimated solid fraction of the ribbons at-gap (*SF*) and solid fraction of the ribbons after elastic recovery (*SF*) in randomized order as conducted at the measurement angle of 70°. Image 1
