# Supplementary material for: Kinetics of elastic recovery in roll compaction
Source: Int J Pharm X. 2024 Nov 14;8:100303. doi: 10.1016/j.ijpx.2024.100303 (PMC11616608; doi:10.1016/j.ijpx.2024.100303)
Supplement: Supplementary file 1 — Table S1: MCC: Process parameters (SCF, S and RS) with the measured ribbon thickness (∆x), the estimated solid fraction of the ribbons at-gap (SF) and solid fraction of the ribbons after elastic recovery (SF) in randomized order as conducted at the measurement angle of 70°. Table S2: MCC+DCPA: Process parameters (SCF, S and RS) with the measured ribbon thickness (∆x), the estimated solid fraction of the ribbons at-gap (SF) and solid fraction of the ribbons after elastic recovery (SF) in randomized order as conducted at the measurement angle of 70°. Table S3: HPC+DCPA: Process parameters (SCF, S and RS) with the measured ribbon thickness (∆x), the estimated solid fraction of the ribbons at-gap (SF) and solid fraction of the ribbons after elastic recovery (SF) in randomized order as conducted at the measurement angle of 70°. [file mmc1.pdf]

Kinetics of elastic recovery in roll compaction: Supplement

Martin Lück[a], Stefan Klinken[a], Peter Kleinebudde[a,*]

[a] Heinrich Heine University Düsseldorf, Faculty of Mathematics and Natural Sciences, Institute of Pharmaceutics and Biopharmaceutics, Universitaetsstrasse 1, 40225 Düsseldorf, Germany

* Corresponding author. E-mail address: kleinebudde@hhu.de

Table S1: MCC: Process parameters (SCF, S and RS) with the measured ribbon thickness ($\Delta\bar{x}$), the estimated solid fraction of the ribbons at-gap (SF_{Mi}) and solid fraction of the ribbons after elastic recovery (SF_{ribbon}) in randomized order as conducted at the measurement angle of 70°.

SCF / kN/cm	S / mm	RS / rpm	$\Delta\bar{x}$ / mm	SF_{Mi} / g/cm³	SF_{ribbon} / g/cm³
7.0	2.30	3.5	2.440	0.856	0.633
10.0	1.48	6.0	1.578	0.994	0.761
7.0	2.31	3.5	2.433	0.855	0.667
10.0	2.98	6.0	3.198	0.908	0.687
4.0	1.50	6.0	1.626	0.782	0.605
10.0	1.54	1.0	1.585	0.990	0.809
4.0	3.00	1.0	3.207	0.714	0.566
10.0	3.00	1.0	3.178	0.907	0.667
7.0	2.31	3.5	2.444	0.855	0.660
4.0	3.00	6.0	3.227	0.714	0.538
4.0	1.52	1.0	1.611	0.780	0.618

Table S2: MCC+DCPA: Process parameters (SCF, S and RS) with the measured ribbon thickness ($\Delta\bar{x}$), the estimated solid fraction of the ribbons at-gap (SF_{Mi}) and solid fraction of the ribbons after elastic recovery (SF_{ribbon}) in randomized order as conducted at the measurement angle of 70°.

SCF / kN/cm	S / mm	RS / rpm	$\Delta\bar{x}$ / mm	SF_{Mi} / g/cm³	SF_{ribbon} / g/cm³
10.0	2.50	4.0	2.648	0.743	0.665
7.0	3.00	6.0	3.089	0.685	0.598
7.0	2.00	6.0	2.056	0.711	0.632
13.0	2.01	2.0	2.069	0.795	0.720
13.0	2.00	6.0	2.077	0.795	0.709
10.0	2.50	4.0	2.578	0.743	0.656
7.0	3.01	2.0	3.115	0.685	0.594
10.0	2.50	4.0	2.573	0.743	0.652
13.0	3.00	6.0	3.104	0.767	0.674
13.0	3.00	2.0	3.104	0.767	0.682
7.0	2.0	2.0	2.025	0.711	0.631

Table S3: HPC+DCPA: Process parameters (SCF, S and RS) with the measured ribbon thickness ($\Delta\bar{x}$), the estimated solid fraction of the ribbons at-gap (SF_{Mi}) and solid fraction of the ribbons after elastic recovery (SF_{ribbon}) in randomized order as conducted at the measurement angle of 70°.

SCF / kN/cm	S / mm	RS / rpm	$\Delta\bar{x}$ / mm	SF_{Mi} / g/cm³	SF_{ribbon} / g/cm³
14.0	3.00	2.0	3.104	0.838	0.754
10.0	2.50	3.0	2.597	0.799	0.724
6.0	3.00	2.0	3.043	0.710	0.669
10.0	2.50	3.0	2.591	0.799	0.728
6.0	1.99	4.0	2.120	0.739	0.672
6.0	3.00	4.0	3.003	0.710	0.667
14.0	3.00	4.0	3.111	0.838	0.750
14.0	2.00	2.0	2.164	0.872	0.760
14.0	2.00	4.0	2.146	0.872	0.762
10.0	2.50	3.0	2.578	0.799	0.726
6.0	2.00	2.0	2.081	0.738	0.677
